# Rationale and design of the RISE-HF trial: sucrosomial iron in heart failure

**DOI:** 10.1016/j.ijcha.2026.101976

**Published:** 2026-07-17

**Authors:** Gabriele Masini, Simona Chiusolo, Mattia Alberti, Riccardo Liga, Gian Giacomo Galeotti, Fabio Lattanzi, Riccardo Morganti, Luna Gargani, Roberto Carnevale, Francesco Violi, Annalisa Castagna, Domenico Girelli, Raffaele De Caterina

**Affiliations:** a University of Pisa and Pisa University Hospital, Cardiology 1 Division, Pisa, Italy; b Section of Statistics, University of Pisa, Italy; c Sapienza University of Rome, Rome, Italy; d Department of Medicine, University of Verona and EuroBloodNet Referral Center for Iron Disorders, Azienda Ospedaliera Universitaria Integrata Verona, Verona, Italy

**Keywords:** Iron deficiency, Sucrosomial iron, Heart failure

## Abstract

**Aims:**

Compared with intravenous iron, oral iron is less expensive, avoids healthcare complexities, and reduces the risk of iron overload. However, conventional oral formulations have not improved clinical outcomes in heart failure (HF) and iron deficiency (ID), largely due to poor absorption. Sucrosomial iron (SI) partially bypasses hepcidin-controlled absorption and showed favorable effects in a small case-control study, warranting further evaluation in a randomized trial.

**Methods:**

The Effect of Oral sucRosomIal Iron on exerciSE Capacity and Quality of Life in Patients With Heart Failure (RISE-HF; NCT06270498) is a randomized, placebo-controlled, double-blind study enrolling 60 patients with HF with left ventricular ejection fraction <50%, ID (transferrin saturation (TSAT) < 20%), ferritin <400 μg/L and hemoglobin (Hb) 10–16 g/dL. Participants are randomized 1:1 to receive SI (Sideral Forte®: sucrosomial iron 30 mg + 70 mg vitamin C) or placebo for 24 weeks, with daily dose tailored to Hb (2 capsules for Hb 10–13.9 g/dL; 1 capsule for Hb 14–16 g/dL). Co-primary endpoints are changes from baseline to week 12 in exercise capacity, assessed by the 6-min walk test (6MWT) distance, and in quality of life, assessed by the Kansas City Cardiomyopathy Questionnaire-12. Secondary endpoints include changes in individual co-primary endpoints at week 24, effects on iron status and oxidative stress biomarkers, N-terminal pro type-B natriuretic peptide and echocardiographic indices of cardiac function, and gastrointestinal tolerability.

**Conclusions:**

RISE-HF will determine whether SI can safely improve exercise capacity, quality of life, and iron status in patients with HF and TSAT-defined ID, justifying a larger multicenter trial.

## Highlights

• RISE-HF evaluates oral sucrosomial iron in heart failure using a transferrin saturation-based definition of iron deficiency.
• The trial tests whether oral sucrosomial iron can improve exercise capacity and quality of life.
• Sucrosomial iron is designed to bypass hepcidin-regulated intestinal absorption.
• RISE-HF integrates mechanistic endpoints, including hepcidin and oxidative stress biomarkers.
• Findings may support oral sucrosomial iron as an alternative to intravenous iron in heart failure.

## 1 Introduction

Iron is essential for a plethora of cellular proteins, including key enzymes involved in energy production. Iron deficiency (ID) impairs mitochondrial bioenergetics [1]. High-energy demand tissues, such as the myocardium, are particularly vulnerable to its detrimental effect [1]. ID reduces contractility in both cardiomyocytes and muscle skeletal cells, and these effects can be prevented by restoring cellular iron content [2]. ID is common in patients with heart failure (HF) with a prevalence of up to 68%, and is associated with worse prognosis, independent of anemia [3].

Intravenous (i.v.) iron therapy in patients with HF with reduced or mildly reduced left ventricular ejection fraction (HFrEF and HFmrEF), and with ID improves symptoms, exercise capacity, quality of life, and reduces HF hospitalizations [4], [5]. Despite its benefit, the use of i.v. iron remains underused due to high cost, administration complexity, and also potential safety concern, particularly with repeated dosing. Therapy with i.v. iron, indeed, releases large amounts of iron into the circulation, exceeding transferrin-binding capacity. This labile, non-transferrin bound iron catalyzes free radical formation, promoting oxidative stress and inflammation [6], [7]. In contrast, treatment with oral iron has not been shown to improve clinical outcomes. In the pharmacological management of chronic HF, where most therapies are oral, i.v. administrations are unusual and logistically complex. Evidence against the efficacy and tolerability of oral iron consists of three small clinical studies. In the Iron Supplementation in Heart Failure Patients With Anemia (IRON-HF) study, patients with HFrEF and ID-anemia were randomized to oral ferrous sulphate (200 mg 3 times daily for 8 weeks), i.v. iron sucrose (200 mg weekly for 5 weeks) and placebo. Ferrous sulphate increased transferrin saturation (TSAT) and ferritin (delta TSAT: 5%; delta ferritin: 103 μg/L) but only i.v. iron increased exercise capacity at 3 months [8]. This study, however, included only 23 patients before the trial was stopped due to slow recruitment and limited funding. In a double-blind randomized controlled trial including 54 patients with HFrEF with ID-anemia, oral ferrous sulphate (200 mg three times a day for 12 weeks) improved TSAT and 6-min walk test (6MWT) distance compared with placebo (TSAT: +30% vs +20%, *p* = 0.008; 6MWTD: 46 m vs −14 m, *p* < 0.001) [9]. In the Iron Repletion Effects on Oxygen Uptake in Heart Failure (IRONOUT-HF) study, 225 patients were randomized to iron polysaccharide (150 mg twice daily for 16 weeks) or placebo. The primary endpoint, change in peak maximum oxygen consumption (pVO_2_) at 16 weeks, did not differ between oral iron and placebo (+23 mL/min vs −2 mL/min; *p* = 0.46), neither did the change of the 6MWT distance, nor N-terminal pro–B-type natriuretic peptide (NT-proBNP) [10]. Iron polysaccharide was associated with a significant, although numerically modest TSAT change (difference in TSAT change between groups 3.3, *p* = 0.03), which was inversely related to baseline hepcidin levels, suggesting that elevated hepcidin limits oral iron absorption (*p* = 0.002 for interaction). Limitations of conventional oral iron preparations include metallic taste and frequent gastrointestinal side effects, such as diarrhea and constipation, reported in up to 40% of patients [11].

**Fig. 1:**
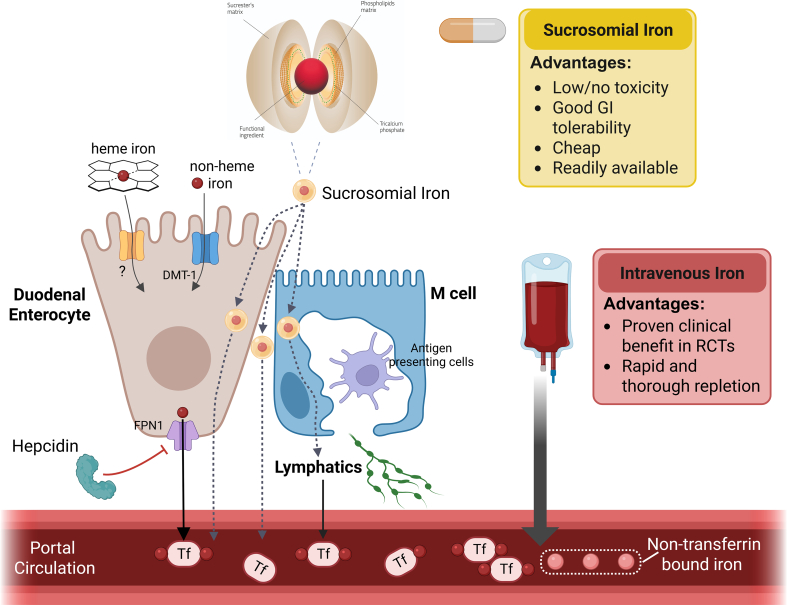
Pathways of iron absorption and supplementation. Due to its unique structure, Sucrosomial Iron® may be absorbed through lymphatic routes, bypassing the ferroportin-hepcidin axis that normally limits intestinal iron uptake. In contrast, intravenous iron releases large amount of elemental iron directly into the blood stream, saturating transferrin capacity and leading to the formation of non-transferrin bound iron, a labile form that promotes oxidative stress. DMT = divalent metal transporter; FPN = ferroportin; Tf = transferrin; M-cell = microfold cell; GI = gastrointestinal; RCTs = randomized controlled trials.

New oral iron formulations have been developed to enhance absorption and minimize gastrointestinal intolerance. Sucrosomial iron (SI) consists of ferric pyrophosphate conveyed by a phospholipid and sucrester matrix, which promotes intestinal absorption through paracellular and transcellular routes (Fig. 1). Preclinical ex vivo and in vitro studies, reviewed in detail elsewhere [12], suggest that SI is absorbed, at least in part, through Divalent Metal Transporter 1-independent pathways involving M cells and the lymphatic circulation. This route may allow a fraction of iron to bypass the ferroportin–hepcidin regulatory axis, consistent with animal studies showing little or no increase in hepatic hepcidin expression following SI supplementation compared with conventional oral salts [13].

SI has been shown to improve iron indices in patients with ID and chronic kidney disease, pregnant women, and, more recently, in patients with chronic HF in a pilot non-randomized study [12], [14]. In that study, Sideral Forte® 1 capsule/day (corresponding to 30 mg SI) administered for 3 months improved 6MWT distance and Kansas City Cardiomyopathy Questionnaire (KCCQ) overall score in 25 patients with HFrEF compared with 25 matched controls, with sustained benefit at 6 months [14]. SI significantly increased ferritin compared with controls (28 ng/mL vs −0.05 ng/mL at 3 months) with good tolerability, since treatment discontinuation occurred in only one patient due to diarrhea. Patients may require more than 3 months of supplementation to achieve a full repletion of iron storage. Extended SI supplementation improves iron indices and exercise capacity in patients with pulmonary hypertension [15]. Compared with ferrous sulphate or iron polysaccharide, SI produces a greater increase of iron indices despite delivering a lower amount of elemental iron [16]. The good bioavailability of SI is attributed to absorption mechanisms involving lymphatic routes that are less or not at all affected by hepcidin regulation. Oral iron replenishes iron stores more slowly than i.v. iron but may offer a safer approach avoiding iron overload and potential pro-oxidant effects, especially in case of repeated infusions. In addition, oral iron is less costly and more practical, as it does not require administration in healthcare facilities. If, therefore, proven effective, this formulation of oral iron may offer significant advantages for the management of HF patients. This set of considerations are the rationale for the “Effect of Oral sucRosomIal Iron on exerciSE Capacity and Quality of Life in Patients With Heart Failure” – RISE-HF Study.

## 2 Study design

**Fig. 2:**
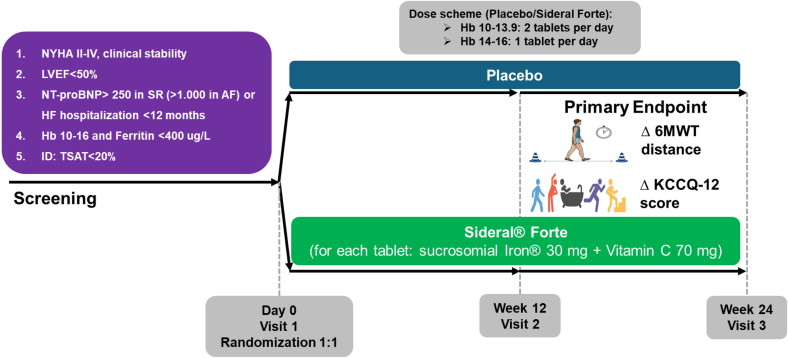
RISE-HF study design. NYHA = New York Heart Association; OMT = optimal medical therapy; LVEF = left ventricular ejection fraction; NT-proBNP=N-terminal pro-B-type natriuretic peptide, pg/mL; SR = sinus rhythm; AF = atrial fibrillation; HF = heart failure; Hb = hemoglobin, g/dL; ID = iron deficiency; TSAT = transferrin saturation; 6MWT = 6 min walk test; KCCQ = Kansas City Cardiomyopathy Questionnaire.

RISE-HF (NCT06270498) is a randomized, double-blind, parallel-group, placebo-controlled, single-center study testing the effect of sucrosomial iron compared with placebo on quality of life and exercise capacity in patients with HF with reduced or mildly reduced left ventricular ejection fraction (LVEF) and ID (Fig. 2). The study has been approved by the Pisa University Hospital Ethics Committee and will be conducted in accordance with the Declaration of Helsinki. All participants will provide written informed consent. The study is registered at ClinicalTrials.gov (NCT06270498). Every subject enrolled will be blinded to the treatment until the study completion. The study is an Investigator-Initiated Study sponsored by the Pisa University Hospital. Enrolment began in June 2024 and is still ongoing.

### 2.1 Objectives and study endpoints

The study primary objective is to investigate the effect of oral SI supplementation (Sideral Forte®, Pharmanutra) compared with placebo on two co-primary endpoints: (1) change in exercise capacity, assessed by 6MWT, and (2) change in quality of life, assessed by KCCQ-12, in patients with HF, LVEF <50% and TSAT <20%. Secondary objectives are to investigate the effect of this oral SI supplementation, compared with placebo, on: biomarkers of iron status (transferrin saturation (TSAT); ferritin and serum iron; soluble transferrin receptor; and hepcidin); echocardiographic measures of left ventricular structure and function (LVEF, LV end-diastolic diameter and volume, LV end-systolic diameter and volume, early to late diastolic transmitral flow velocity (E/A) ratio, E/e’, left atrial volume index, systolic pulmonary artery pressure); N-terminal pro-B-type natriuretic peptide (NT-proBNP); time to heart failure hospitalization or death; marker of oxidative stress (serum F_2_-isoprostanes); phosphate metabolism biomarkers (phosphate and fibroblast growth factor-23). Finally, the study will determine the gastrointestinal tolerability and safety profile of the tested oral SI supplementation.

### 2.2 Study participants

Eligible patients are those with chronic HF, New York Heart Association (NYHA) functional class II-IV, clinically stable for at least 4 weeks, with a reduced or mildly reduced LVEF<50%, TSAT<20%, ferritin <400 μg/L, and hemoglobin 10–16 g/dL. Detailed eligibility criteria are shown in Table 1.

**Table 1:** Eligibility criteria of the RISE-HF study.

**Inclusion criteria**
Chronic HF (NYHA functional class II–IV) patients, on optimal medical therapy, and clinically stable for at least 4 weeks with no dose changes of HF drugs
LVEF <50% at screening visit (historical value can be used if performed within 6 months of screening visit)
Either a documented hospitalization for HF in the previous 12 months or an elevated NT-proBNP: >250 pg/mL (or BNP >75 pg/mL) for patients in sinus rhythm; >1000 pg/mL (or BNP >400 pg/mL) for patients in atrial fibrillation
Transferrin saturation < 20%
Hemoglobin 10.0–16.0 g/dL
Rapid iron repletion with intravenous iron is not considered a clinical necessity by physicians after reviewing patient medical record (if anemia is present, its grade is no more than mild)
Age ≥ 18 years, male and female
Willingness to provide informed consent
Subjects who decide to use single or dual contraceptive methods to avoid conceiving during the study period

**Exclusion criteria**
Neuromuscular, orthopedic or other non-cardiac condition that prevents the patient from exercise testing
Exercise training program in the previous 3 months, or planned in the next 3 months
Recent (<3 month) acute coronary syndrome, coronary artery bypass surgery, percutaneous coronary interventions, transient ischemic attack, or stroke
Severe valvular disease, hypertrophic obstructive cardiomyopathy, restrictive or constrictive cardiomyopathy, acute myocarditis
Atrial fibrillation or flutter with a ventricular response rate of >100 beats per minute at rest
Temperature > 38 °C (oral or equivalent) or active infection as defined by current use of oral or intravenous antimicrobial agents
Need for blood transfusion within the last month
Hb < 10 g/dL or Hb > 16 g/dL
Rapid iron repletion with intravenous iron is considered a clinical necessity by physicians after reviewing patient medical record
Documented active gastrointestinal bleeding
Oral iron, i.v. iron or erythropoietin stimulating agent within the last 3 months
Estimate glomerular filtration rate ≤ 15 mL/min or on hemodialysis
Chronic liver disease and/or alanine transaminase or aspartate transaminase above 3 times the upper limit of the normal range
Active cancer
Evidence of iron overload (ferritin >400 ng/mL)
Hypersensitivity to any of the study products or known severe allergies
Participation in another study
Low body weight (<35 kg)
Known or expected pregnancy in the next 6 months
Need for forbidden medications
Breastfeeding
Consumption of iron-rich foods or any food that alter iron absorption (i.e., food rich in vitamin C) due to dietary requirements
Any pathological condition or disease associated with a reduction or an impairment of intestinal iron absorption (i.e., prior gastrectomy, atrophic gastritis, bariatric surgery, coeliac disease)

HF = heart failure; NYHA = New York Heart association; LVEF = left ventricular ejection fraction; NT-proBNP = N-terminal pro-type-B natriuretic peptide.

### 2.3 Randomization and allocation concealment

Patients are randomly assigned in a 1:1 ratio to the active arm or standard of care using an online, secure randomization software. Block randomization is applied, stratified by hemoglobin strata (10–13.9 vs 14–16) as the grade of anemia may influence clinical response to iron supplementation [17].

### 2.4 Study intervention

**Intervention arm.** Sideral® Forte is registered as a dietary supplement containing Sucrosomial Iron® 30 mg per capsule, a patented formulation that allows the mineral to pass through the stomach intact and be absorbed in the intestine, thus avoiding stomach irritation and discomfort typically associated with other oral iron formulations. The addition of Vitamin C (70 mg per capsule) helps promote iron absorption. Sideral® Forte will be administered orally once a day for 6 months on top of the best medical therapy. The dose regimen of SI will be calculated according to the hemoglobin levels at baseline evaluation: 1 capsule once a day (30 mg of SI) for 24 weeks if hemoglobin 14–16 g/dL; 2 capsules once a day (60 mg of SI) for 24 weeks if hemoglobin 10–13.9 g/dL. This dosing scheme reflects patient's iron need in accordance with previous studies [18]. Patients will be advised to take both capsules in the morning, away from meals.

**Standard of care.** Placebo capsules are identical in shape, form and color to SI capsules; they will contain the same components of SideraL Forte except for Sucrosomial Iron® and Vitamin C. Active supplement and placebo capsules will be provided by Pharmanutra free of charge in sufficient quantities for daily dosing for each patient throughout the study. Placebo capsules will be administered orally once a day for 24 weeks according to the same dose scheme as in the intervention arm.

Study participants will be treated with optimal HF therapies as recommended by international guidelines, and will continue to receive standard HF care according to international guidelines after study completion, with post-study management independent of study investigators to preserve the integrity of the blinded study design.

**Safety Considerations.** No harm is anticipated for patients participating in this study, as SI is a dietary supplement with a good safety and tolerability profile. The most common adverse effects of oral iron supplementation are gastrointestinal, and include constipation, diarrhea, nausea, vomiting, dark stools, and abdominal pain. Gastrointestinal adverse reactions are dose-related and typically decrease over time. Gastrointestinal adverse events will be monitored throughout the study. In the event of persistent symptoms, dose reduction or treatment discontinuation is allowed at the investigator's discretion, in accordance with the study protocol. In a previous small case-control study, of 25 patients with HFrEF treated with SI, oral supplementation was discontinued in only one patient due to diarrhea [14]. Benefits and advantages of Sideral® Forte include high intestinal absorption, excellent tolerability, excellent palatability (neutral taste), and a gluten-free formulation. SI is not expected to interfere with the absorption or pharmacokinetics of concomitant cardiovascular medications, and no clinically relevant drug–drug interactions have been reported.

**Subject Withdrawal and intervention modifications.** Subjects may voluntarily withdraw from study participation at any time without providing a reason. Subjects may be withdrawn because of: the appearance of a new health condition suspected to require care; moderate or severe anemia (hemoglobin <10.0 g/dL) for which a rapid iron repletion with intravenous iron is considered a clinical necessity; hemoglobin >16.0 g/dL; need for medications or treatments prohibited by the protocol; unacceptable adverse effects; refusal to continue study treatment; at investigator's discretion if in the subjects' best interest. The following therapies or treatments will be prohibited and not be provided concurrent with the study treatment: other oral or any i.v. iron formulations; erythropoietin or analogues; blood transfusions; immunosuppressive or myelosuppressive therapies; surgery that may result in significant blood loss (>1 g/dL hemoglobin); kidney dialysis; heart transplant. At any of these occurrences' participants will be withdrawn from the study.

### 2.5 Investigation plan and study endpoints

A visual schedule of assessments throughout the study is provided in Table 1**, Supplementary Material**. Co-primary endpoints will consist of (1) the 6MWT distance change, expressed in meters; and (2) the difference in KCCQ-12 overall score change from baseline to week 12 between SI and placebo group. Secondary endpoints are reported in detail in Table 2.

**Table 2:** Secondary endpoints of the RISE-HF study.

Proportion of patients with a 15-m improvement in 6MWT distance (responders), from baseline to week 12
Proportion of patients with a 5-point improvement in KCCQ-12 overall score (responders), from baseline to week 12
Difference in oxidative stress markers (F_2_-isoprostanes, Soluble NOX2–derived peptide and H_2_O_2_) from randomization to week 12
Difference in serum phosphate and intact FGF-23 from randomization to week 12
Difference in soluble receptor of transferrin and hepcidin from randomization to week 12
Difference in iron indices (TSAT, ferritin and serum iron) from randomization to week 12
Difference in NT-proBNP from randomization to week 12
Difference in echocardiographic parameters (LVEF, LV end diastolic diameter and volume, LV end systolic diameter and volume, mitral E/A, E/e’, left atrial volume index, systolic pulmonary artery pressure) from randomization to week 12
Time to death or to first HF hospitalization from randomization to week 12
Proportion of patients with a 15-m improvement in 6MWT distance (responders), from baseline to week 24
Proportion of patients with a 5-point improvement in KCCQ-12 overall score (responders), from baseline to week 24
Difference in 6MWT distance change, expressed as meters, from baseline to week 24
Difference in KCCQ-12 overall score change, from baseline to week 24
Difference in oxidative stress markers (F_2_-isoprostanes, Soluble NOX2–derived peptide and H_2_O_2_) from randomization to week 24
Difference in serum phosphate and intact FGF-23 from randomization to week 24
Difference in soluble receptor of transferrin and hepcidin from randomization to week 24
Difference in NT-proBNP from randomization to week 24
Difference in iron indices (TSAT, ferritin and serum iron) from randomization to week 24
Number of any adverse events
Number of allergic reactions
Time to death or to first HF hospitalization from randomization to week 24

6MWT = six-minute walk test; KCCQ = Kansas City Cardiomyopathy Questionnaire; NT-proBNP = N-terminal pro-type-B natriuretic peptide; TSAT = transferrin saturation; LVEF = left ventricular ejection fraction; FGF-23 = Fibroblast Growth Factor-23.

### 2.6 Sample size estimation and statistical analyses

No data on the effect of SI on exercise capacity and quality of life from randomized clinical studies are available. In a case-control pilot study, 25 patients treated with 30 mg/day of SI for 3 months showed a statistically significant between-group improvement of ∼12 m in 6MWT and ∼ 4 points in KCCQ-12 score at week 12, compared with age- and sex-matched controls [14]. While these findings support a potential benefit of SI, prior studies in HFrEF with iron deficiency have established that a 15-m increase in 6MWT distance and a 5-point increase in KCCQ-12 score represent the minimal clinically important differences [5], [19]. Therefore, the present study is powered to detect these more conservative thresholds. A decline in exercise performance and quality of life was apparent in the control group, consistent with data from previous studies testing i.v. iron in chronic HF [20]. In the present study, active treatment consists of SI according to patients' hemoglobin (30 mg, 60 mg), rather than a fixed dose of 30 mg, as in the previous case-control study [14]. Therefore, we expect a greater improvement in 6MWT distance and KCCQ-12 in the SI group. Assuming a pooled standard deviation of 15 m for 6MWT distance and 4 points for the KCCQ-12 score, and no changes in the primary endpoints in the placebo arm, we estimate that a total sample size of 60 patients (30 per group) would be required to detect a significant between-group difference of 15 m in 6MWT distance and 5 points in KCCQ-12 score, with 90% power, a significance level of 0.025 for each co-primary endpoint (Bonferroni-corrected), and a dropout rate of 10%. The RISE-HF trial includes two co-primary endpoints: (1) change in 6MWT distance from baseline to week 12, and (2) change in KCCQ-12 overall summary score from baseline to week 12. Each endpoint will be analyzed independently using an analysis of covariance (ANCOVA) model adjusted for the corresponding baseline value. Sensitivity analyses using rank-based ANCOVA with Hodges-Lehmann estimators will be conducted to ensure robustness to deviations from normality and outliers. To control the family-wise type I error rate at the prespecified two-sided α = 0.05 across the two co-primary endpoints, the Hochberg step-up procedure will be used. The study will meet its prespecified objective if either: (1) both two-sided *P* values are ≤0.05; or (2) the smaller two-sided P value is ≤0.025. Missing data will be handled using multiple imputation assuming random missing data, and sensitivity analyses without imputation will be conducted to assess the robustness of the results of the primary analysis. Secondary endpoints will be tested only if at least one co-primary endpoint is significant under the co-primary Hochberg family-wise test. A responder analysis will be performed using an improvement of 15 m in 6MWT distance and 5 points in KCCQ-12. The proportion of responders will be compared between groups using logistic regression adjusted for baseline values. Strong control of the family-wise type I error will be applied to the family of clinical secondary endpoints using Hochberg procedure. The clinical family comprises: 6MWT change at Week 24; KCCQ-12 change at Week 24; 6MWT responder (≥15 m increase); KCCQ-12 responder (≥5-point increase). All other secondary endpoints will be considered exploratory and reported with nominal *p*-values and confidence intervals.

## 3 Discussion

International guidelines on HF recommend i.v. iron in patients with HF with reduced LVEF and ID, regardless of the presence of anemia, to improve symptoms and quality of life [21], [22]. The European Society of Cardiology guidelines also recommend i.v. iron in patients with reduced or mildly reduced HF to reduce the risk of HF hospitalization (class of recommendation IIa, level of evidence A) [22]. Limited and inconclusive evidence support iron therapy in patients with heart failure and preserved LVEF. Despite the favorable effects of i.v. iron, patients with HF are rarely screened for ID, and i.v. supplementation is poorly implemented in clinical practice. In a registry including more than 21,000 patients with chronic HF, only 27% were screened for ID and, of those with ID, only 19% were treated with i.v. iron [23]. Importantly, registry data show that the use of oral iron is higher compared with the use of i.v. iron (28% vs 13%) [24]. In many European countries, i.v. iron approved indication is when oral iron treatment is ineffective, not tolerated, or rapid iron repletion is considered a clinical necessity, leaving the choice between i.v. or oral treatment to the clinicians' judgment. Based on estimated iron loss, most patients require more than one infusion of i.v. iron to become iron-replete [24]. In clinical practice, patients admitted for acute HF with ID usually receive a single dose of i.v. iron before discharge, while subsequent doses are seldom administered for practical reasons. Iron infusion protocols advocate patients to be monitored for adverse effects for at least 30 min after the injection, thus requiring iron infusions in healthcare facilities. In addition, ferric carboxymaltose, the most used i.v. iron product, has been associated with a higher risk of hypophosphatemia, which may impair bone health [25].

New oral iron formulations, such as SI, may have higher intestinal absorption than conventional oral formulations (e.g., ferrous sulphate or ferrous gluconate) tested in past studies, and may better restore iron stores [26], significantly improving symptoms and quality of life. This hypothesis has prompted new clinical studies assessing SI. So far, evidence supporting a clinical benefit of new oral formulation in patients with HF is limited to one small case-control study testing SI [14]. Characteristics of ongoing randomized control studies, including the present study, testing the effect of SI in patients with HF, are summarized in Table 3.

**Table 3:** Studies assessing sucrosomial iron in heart failure.

Name	PREFER-HF	IVOFER-HF	RISE-HF	VICTORID-HF
Identifier	NCT03833336	2017-005053-37	NCT06270498	NCT05702970
Start year	2017	2019	2024	2024
N	72	572	60	258
LVEF, %	>45	<45	<50	≤45
NT-proBNP, pg/mL	>400	>400	>250	>400
Hemoglobin, g/dL	Not specified	<13 for men; <12 for women	10–16	9.5–13.5
Definition of ID	F < 100 or TSAT<20 if F 100–300	F < 100 or TSAT<20 if F 100–300	TSAT<20 and F < 400	F < 100 or TSAT <20 if F 100–300; 25OH-vitD <50
Intervention(s)	FCM 500–1000 mg (based on Hb and body weight) at week 0,6,12,24(1); Ferroglycine sulphate 2 × 100 mg until week 24(2); Sucrosomial iron 2 × 30 mg until week 24(3)	FCM or ferroglycine sulphate or liposome iron	Sucrosomial iron 30–60 mg (based on Hb) od until week 24	Sucrosomial iron 60 mg od until week 24 (1); Sucrosomial iron 60 mg od + vit D 100.000 UI load at time 0 then 2.000 UI daily until week 24
Comparator	Placebo	Placebo	Placebo	Placebo
Primary endpoints	6MWT distance At week 24	6MWT distance At week 24	6MWT distance and KCCQ at week 12	6MWT distance at week 24
Secondary endpoints	HYHA KCCQ Rate of HF or CV hospitalizations Time to all-cause and CV-death	HYHA KCCQ HF or CV hospitalizations all-cause and CV-death	6MWT distance at week 24 KCCQ at week 24 TSAT, ferritin, serum iron, hepcidin, sTfR NT-proBNP Echo measures of cardiac function F2-isoprostanes Phosphate and FDG-25 Tim to death of first HF hospitalization	KCCQ NYHA Estimated GFR Survival days out of hospital Hospitalizations Mortality Echo measures of cardiac function Calcium and phosphate iFGF-23 Fractures
Current status^⁎^	Completed (no results available)	Ongoing	Rectruiting	Not recruiting yet

LVEF = left ventricular ejection fraction; NT-proBNP = N-terminal pro-type-B natriuretic peptide; ID = iron deficiency; F = ferritin, μg/L; TSAT = transferrin saturation, %; 25OH-vitD = 25-hydroxyvitamin D; FCM = ferric carboxymaltose; Hb = hemoglobin; iFGF-23 = intact fibroblast growth factor-23 6MWT = six-minute walk test; NYHA = New York Heart association; KCCQ = Kansas City Cardiomyopathy Questionnaire; HF = heart failure; CV = cardiovascular; sTfR = soluble transferrin receptor; Echo = echocardiographic; GFR = glomerular filtration rate. ^⁎^according to data available on trials registries website.

The RISE-HF study differs from other studies in several key points. This is the first study to use a definition of ID based on TSAT <20%. Almost all studies assessing iron therapy in HF used ferritin-based definitions (ferritin <100 μg/L or TSAT <20% if ferritin 100–299 μg/L) [7], as supported by international guidelines [21], [22]. Compared to ferritin-based definitions, TSAT <20% predicts better ID, defined by bone marrow biopsy, and is associated with a worse outcome [3]. More importantly, in randomized clinical trials testing i.v. iron, TSAT but not ferritin predicts a favorable clinical response, and adoption of TSAT <20% to define ID is now advocated by HF experts [27]. Patients with ferritin >300 μg/L who are not considered iron-deficient under European Society of Cardiology guidelines criteria, may still have true ID and benefit from iron therapy [3], [27]. In the present study we adopted a ferritin cut-off of 400 μg/L as an exclusion criteria, in line with a previous trial on i.v. iron [28].

The RISE-HF study will also address mechanistic gaps in iron metabolism providing data on the influence of hepcidin on SI absorption, as hepcidin will be measured at baseline and after 3 and 6 months in all patients. Some studies support the concept that iron absorption is more effective with intermittent oral dosing (every other day) compared with daily dosing [29]. After the oral iron intake, hepcidin production increases for at least 24 h, preventing further iron absorption from the next dose. However, data supporting intermittent dosing comes from studies testing ferrous sulphate. Since SI absorption may, at least in part, bypass hepcidin-mediated regulation, we anticipate that daily dosing will have little or no effect on SI efficacy.

The RISE-HF study will test the hypothesis that SI has little or no pro-oxidant effect, which is a concern of i.v. iron therapy. Supplementation with i.v. iron releases large amounts of iron into the blood, exceeding transferrin-binding capacity. This non-transferrin-bound labile iron promotes free radical generation and inflammation [6], [7]. I.v. iron may induce oxidative stress within tubular renal cells potentially promoting chronic kidney disease [30]. Iron may enter cardiomyocytes through specific pathways that expose the myocardium to iron overload after repetitive iron infusions [31]. Evidence of pro-oxidant effects from oral iron is limited to gastrointestinal oxidative stress with ferrous salts, which contributes to its poor gastrointestinal tolerability [6], while data on other oral formulations are scarce. In the RISE-HF study, oxidative stress will be assessed using serum F2-isoprostanes, soluble NOX2-derived peptide, and hydrogen peroxide (H_2_O_2_), which are highly accurate markers of in vivo lipid peroxidation [32].

Unlike other studies, the RISE-HF does not include an i.v. iron arm, which is the current standard of care. We acknowledge this as a study limitation. However, i.v. and oral supplementation should not necessarily be viewed as mutually exclusive. Oral iron may replenish stores more slowly and to a lesser extent than i.v. iron, potentially limiting comparable outcome benefits. Nonetheless, oral iron may avoid iron overload in patients receiving repeated i.v. doses and improve adherence in those with limited access to healthcare. A combined approach, consisting of i.v. iron, administered before discharge in case of patients admitted for acute HF, followed by oral iron for at least 3 months, could provide the optimal balance of efficacy and safety. Moreover, in stable patients with mild-ID and no ID-anemia, which seems to obtain small or no benefit from i.v. iron [17], SI for at least 3 months may suffice to replete iron stores, with iron status reassessment after 3 months to determine the need for further therapy.

The RISE-HF trial is designed as a proof-of-concept study aimed at generating preliminary evidence. If successful, it could pave the way for larger multicenter trials and ultimately broaden therapeutic options beyond i.v. formulations, increasing the implementation of iron repletion in clinical practice.

## 4 Conclusions

In contrast to iron salt-based oral formulations, new oral iron products such as SI may offer better bioavailability and gastrointestinal tolerability, improve iron stores and potentially yield clinical benefits. The RISE-HF study, a randomized placebo-controlled trial, will evaluate whether SI improves exercise capacity and quality of life in patients with chronic HF, LVEF<50%, and TSAT-defined ID. Secondary analysis will examine the influence of hepcidin and whether SI induces oxidative stress, a concern with i.v. iron. These data will inform future strategies for managing ID in HF, potentially supporting larger multicenter studies on SI.

## Authorship statement

All authors take responsibility for all aspects of reliability and freedom from bias of the data presented and their discussed interpretation.

## CRediT authorship contribution statement

**Gabriele Masini:** Writing – review & editing, Writing – original draft, Methodology, Investigation, Conceptualization. **Simona Chiusolo:** Writing – review & editing, Investigation, Conceptualization. **Mattia Alberti:** Writing – review & editing, Investigation. **Riccardo Liga:** Writing – review & editing, Investigation. **Gian Giacomo Galeotti:** Writing – review & editing, Investigation. **Fabio Lattanzi:** Writing – review & editing, Investigation. **Riccardo Morganti:** Writing – review & editing, Methodology. **Luna Gargani:** Writing – review & editing. **Roberto Carnevale:** Writing – review & editing, Methodology, Investigation. **Francesco Violi:** Writing – review & editing, Supervision. **Annalisa Castagna:** Writing – review & editing, Methodology, Investigation. **Domenico Girelli:** Writing – review & editing, Supervision, Methodology. **Raffaele De Caterina:** Writing – review & editing, Supervision, Project administration, Methodology, Conceptualization.

## Funding

The study is an Investigator-initiated study sponsored by the Pisa University Hospital. Pisa University Hospital has received funds from Pharmanutra S.p.A. to cover some costs related to the conduction of the study, as well as the study medication and the corresponding placebo. However, Pharmanutra S.p.a. will have no involvement in the conduct of the study.

## Declaration of competing interest

GM has consulted for Pharmacosmos. All the other authors have no conflict of interests in relation to this study.

## Appendix A Supplementary data

Supplementary Table 1. Schedule of study visits and assessments in the RISE-HF trial.
