# Supplementary material for: Rationale and design of the RISE-HF trial: sucrosomial iron in heart failure
Source: Int J Cardiol Heart Vasc. 2026 Jul 17;65:101976. doi: 10.1016/j.ijcha.2026.101976 (PMC13400243; doi:10.1016/j.ijcha.2026.101976)
Supplement: Supplementary file 1 — Supplementary Table 1. Schedule of study visits and assessments in the RISE-HF trial. [file mmc1.docx]

**Rationale and Design of the RISE-HF Trial: Sucrosomial Iron in Heart Failure**

Gabriele Masini, MD,^1^ Simona Chiusolo, MD,^1^ Mattia Alberti, MD,^1^ Riccardo Liga, MD, PhD,^1^ Gian Giacomo Galeotti, MD,^1^ Fabio Lattanzi, MD,^1^ Riccardo Morganti, MS,^2^ Luna Gargani, MD, PhD,^1^ Roberto Carnevale, MD, ,^3^ Francesco Violi, MD,^3^ Annalisa Castagna, MS,^4^ Domenico Girelli, MD, PhD^4^ and Raffaele De Caterina, MD, PhD^1^

**ONLINE SUPPLEMENTARY MATERIAL**

| **Table 1. Components of study visit assessment** | | | | |
| --- | --- | --- | --- | --- |
|  | **V0**  **Screening** | **V1**  **Baseline + Randomization (< 2 weeks from screening)** | **V2**  **Week 12 from randomization** | **V3**  **Week 24 from randomization (end of treatment)** |
| **Inclusion/Exclusion criteria** | x |  |  |  |
| **Written informed consent** | x |  |  |  |
| **Medical/Interim History** | x | x | x | x |
| **Clinical examination** | x | x | x | x |
| **NYHA class** | x | x | x | x |
| **Medications review** | x | x | x | x |
| **ECG** |  | x | x | x |
| **Echocardiography** |  | x | x | x |
| **6-minute walk test** |  | x | x | x |
| **KCCQ-12 questionnaire** |  | x | x | x |
| **Blood sample** | x | x | x | x |
| **NT-proBNP/BNP** | x | x^1^ | x | x |
| **TSAT** | x | x^1^ | x | x |
| **Ferritin** | x | x^1^ | x | x |
| **Serum iron** | x | x^1^ | x | x |
| **sTfR, Hepcidin** |  | x | x | x |
| **Complete blood count, hemoglobin, sodium, potassium, chloride, creatinine, glucose, ALT, AST, and total bilirubin** | x | x^1^ | x | x |
| **Phosphate, FGF-23** |  | x | x | x |
| **Serum F2-isoPs, NOX2,H_2_O_2_** |  | x | x | x |
| **Gastrointestinal side effects** |  |  | x | x |
| **Other side effects** |  |  | x | x |
| **β-hCG analysis on urine (dipstik)** |  | x |  |  |
| **Dispensing SiderAL® Forte/Placebo** |  | x | x |  |

Randomization should occur within 2 weeks of screening blood tests. Bloods will be collected either during the study visit or in advance of visit (within 2 weeks). Results must be available prior to randomization (to comply with dose scheme).^1^use values from screening phase if available. NYHA= New York Heart Association; ECG= electrocardiogram; KCCQ= Kansas City Cardiomyopathy Questionnaire; NT-proBNP/BNP= N-terminal pro-B-type natriuretic peptide/ B-type natriuretic peptide; TSAT=transferrin saturation; sTfR= soluble transferrin receptor; ALT= alanine transaminase; AST=aspartate transaminase; FGF= fibroblast growth factor; F2-isoPs= F2-isoprostanes; sNOX2-dp=soluble NOX2–derived peptide; β-hCG=beta human chorionic gonadotropin.
